# Effect of *Lactobacillus plantarum* PS128 on neuromuscular efficiency after a half-marathon

**DOI:** 10.3389/fphys.2023.1254985

**Published:** 2023-11-30

**Authors:** Chia-Hsien Yu, Chang-Chi Lai, Jing-Hsuan Chen, I-Cheng Chen, Hsia-Ling Tai, Szu-Kai Fu

**Affiliations:** ^1^ Graduate Institute of Sports Training, College of Kinesiology, University of Taipei, Taipei, Taiwan; ^2^ Department of Exercise and Health Sciences, College of Kinesiology, University of Taipei, Taipei, Taiwan; ^3^ Office of Physical Education, Tamkang University, New Taipei, Taiwan; ^4^ Department of Physical Education, College of Science, University of Taipei, Taipei, Taiwan

**Keywords:** *Lactobacillus plantarum* PS128, median power frequency, spectrum analysis, electromyography, muscle fatigue

## Abstract

**Introduction:** *Lactobacillus plantarum* PS128 (PS128) could be considered an antioxidant supplement to reduce muscle fatigue and improve exercise capacity recovery after vigorous exercise.

**Purpose:** The purpose of this study is to investigate the effect of PS128 on muscle fatigue and electromyography (EMG) activity after a half-marathon (HM).

**Methods:** The experimental design used a repeated-measures design with a double-blind approach. The participants either took two capsules of PS128 for 4 weeks as the PS128 group (PSG, *n* = 8) or took two capsules of a placebo for 4 weeks as the placebo group (PLG, *n* = 8) to ensure counterbalancing. The time points of the maximal voluntary isometric contraction (MVIC) and EMG activity test were set before probiotics were taken (baseline), 48 h before HM (Pre), and immediately at 0 h, 3 h, 24 h, 48 h, 72 h, and 96 h after HM.

**Results:** EMG activity included median power frequency (MDF), integrated EMG (iEMG), and neuromuscular efficiency (peak torque/iEMG). The MVICs of knee extensors, analyzed by using an isokinetic dynamometer, showed a decrease from the Pre to 0 h (*p* = 0.0001), 3 h (*p* < 0.0001), 24 h (*p* < 0.0001), 48 h (*p* < 0.0001), 72 h (*p* = 0.0002), and 96 h (*p* = 0.0408) time points in the PLG. Sidak’s multiple comparisons tests showed that the PLG was significantly lower than the PSG at 0 h (*p* = 0.0173), 3 h (*p* < 0.0001), 24 h (*p* < 0.0001), 48 h (*p* < 0.0001), 72 h (*p* < 0.0001), and 96 h (*p* = 0.0004) time points. The MDF of vastus medialis oblique (VMO) in the PLG was significantly decreased 24 h after HM and significantly lower than that in the PSG at all times points after HM. The iEMG of VMO in the PLG was significantly decreased 48 h after HM and significantly lower than that in the PSG at 0 h, 3 h, 24 h, 48 h, and 72 h after HM.

**Conclusion:** The PS128 supplementation may prevent the decrease in MDF, iEMG, and peak torque after vigorous exercise. Recreational runners may consider implementing a probiotic supplementation regimen as a potential strategy to mitigate muscle fatigue following HM.

## 1 Introduction

Long-distance running, like marathons and half-marathons (HMs), is incredibly demanding due to its prolonged exertion, eccentric movements, and high intensity (Hill et al., 2014). Post-exercise muscle fatigue is the result of complex factors involving the terminal end of the alpha motor neuron within skeletal muscles, which remain incompletely understood. This fatigue often leads to a significant decrease in muscle performance lasting several days (Petersen et al., 2007; García-Manso et al., 2011). To aid recovery and promote athlete wellbeing, strategies are needed to expedite their return to regular training without increasing injury or illness risks (Elias et al., 2012; Delextrat et al., 2013). Muscle fatigue is a temporary reduction in the maximum force or power that a muscle can produce, which is caused by physical exercise (Mackey et al., 2008; Carroll et al., 2017). This condition can cause a decrease in muscle function and joint stability (Rowlands et al., 2001; Paschalis et al., 2007), which can negatively impact exercise performance (Cheung et al., 2003; Burt and Twist, 2011). The factors believed to cause muscle fatigue include the accumulation of metabolites in the muscle and inadequate motor command within the neuromuscular system (Enoka and Duchateau, 2008).

Surface electromyography (EMG) is commonly used to evaluate muscle fatigue, as it can non-invasively and quantitatively measure myoelectrical activity in real time. Different indexes have been proposed to analyze EMG signals, including the median frequency (MDF), mean frequency (MNF), root mean square (RMS) for integrated EMG (iEMG), fractal dimension, and average rectified value (De Luca, 1984; Kahl and Hofmann, 2016; Hou et al., 2021). The shift in MDF primarily reflected biochemical changes in type 2 muscle fibers, indicating fatigue in the fast-twitch motor units (Gerdle and Fugl-Meyer, 1992). The functional state of the neuromuscular system could be described in terms of its capacity to generate a specific level of muscle activation, known as neuromuscular efficiency (NME) (David and Mora, 2008). The amplitude of the bioelectric activity in the muscles could be evaluated through EMG signals, which provide insights into both peripheral and central strategies employed by the neuromuscular system, especially during muscle contractions and force production (Farina et al., 2004). In individuals with type 2 diabetes, training-related enhancement in NME was associated with higher peak torques, while the neural signals controlling muscle activation, indicated by RMS, showed minimal change. This implies that improved NME may be influenced by the central nervous system (CNS) (Gabriel et al., 2006).

Peripheral fatigue involves a decrease in neuromuscular junction effectiveness and metabolic changes within muscles (Ament and Verkerke, 2009; Korzeniewski, 2019). Muscular fatigue likely results from metabolite accumulation due to reactive oxygen species (ROS), including inorganic phosphates, calcium ions, lactate, ADP, magnesium, and glycogen depletion, disrupting homeostasis (Myburgh, 2004). This type of fatigue is particularly pronounced during high-intensity exercise, as the buildup of metabolites can disrupt the interaction between actin and myosin cross-bridges. Consequently, this leads to a reduction in the activity of the ATPase enzyme, which is directly related to the contraction rate. The metabolic environment fostering peripheral and central fatigue is characterized by acidosis with low pH levels and an accumulation of phosphates. These factors work in synergy, reducing the muscle fiber’s ability to generate force (Woodward and Debold, 2018). Peripheral fatigue, linked to homeostasis, arises significantly when surpassing the onset of blood lactate accumulation (OBLA) during exercise, leading to the buildup of substances like lactate, hydrogen, and ammonia in the blood. Exercise-induced fatigue may also affect the action potential speed along the sarcolemma due to changes in the muscle fiber environment. Increased release of potassium ions (K+) from muscle fibers is another critical factor, obstructing tubular action potential within the T-tubules and reducing force output due to excitation–contraction coupling decline (Ament and Verkerke, 2009).

*Lactobacillus plantarum* PS128 (PS128) is a beneficial probiotic known to enhance the immune system, reduce inflammation, and modulate immune responses (W.H. Liu et al., 2015). It has also shown promise in alleviating anxiety-like behaviors, potentially aiding in the management of neuropsychiatric disorders (W. H. Liu et al., 2016). Recent research has emphasized its efficacy in reducing oxidative stress and muscle damage in endurance athletes after prolonged exercise (Huang et al., 2019)*.* During exercise, ROS are produced in active muscles, leading to the formation of products such as lipid peroxides and oxidized proteins (Davies et al., 1982; Diaz et al., 1993). These ROS products might play a crucial role in the development of fatigue, particularly during moderate-to-intense exercise such as running (Parker et al., 2018; Thirupathi et al., 2021). Using PS128 as an antioxidant might reduce muscle fatigue and improve exercise capacity recovery after HM. This performance improvement is thought to be due to an increase in antioxidant capacity and a reduction in muscle damage. (Huang et al., 2019; Fu et al., 2021).

This study aimed to investigate how PS128 affects NME in the context of exercise. It was hypothesized that after HM, PS128 intake could a) prevent a decrease in force and iEMG in knee extensors, thus maintaining NME, and b) prevent a reduction in MDF, indicating a lesser reduction in the recruitment of lower fast-twitch muscles.

## 2 Materials and methods

### 2.1 Study design

**FIGURE 1:**
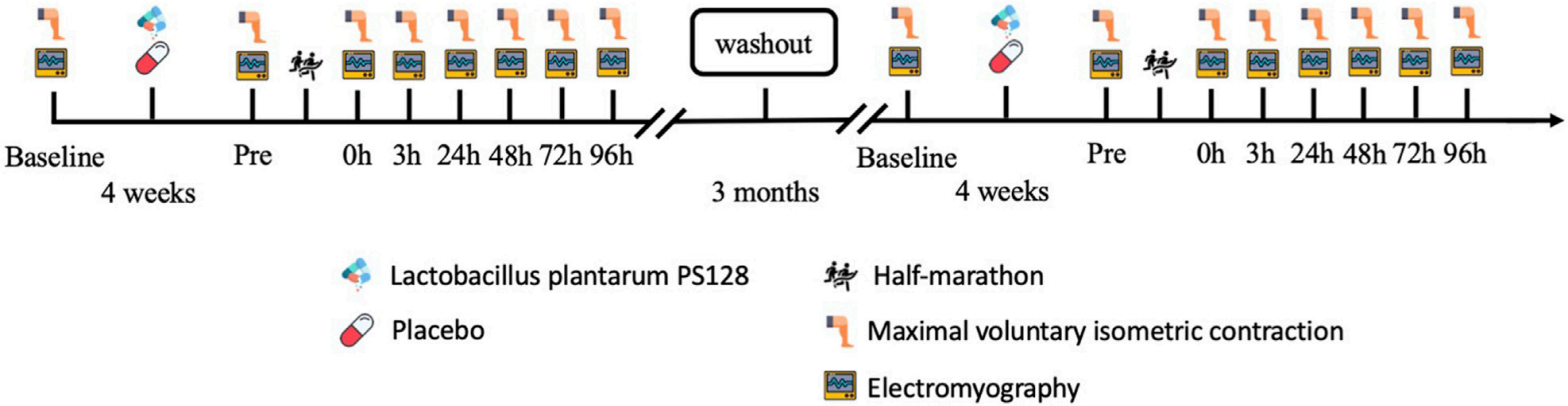
Experimental design flowchart.

A double-blind, repeated-measures design was used to evaluate the effectiveness of PS128 in preventing the reduction of iEMG, MDF, and NME following HM and its potential to improve muscle fatigue. The participants took two capsules of PS128 before breakfast and dinner for 4 weeks as the PS128 group (PSG, *n* = 8) or took two capsules of placebo for 4 weeks as the placebo group (PLG, *n* = 8) to ensure counterbalancing. After all the experimental protocols, they were going to execute a 3-month washout period. Subsequently, the two groups switched capsules and executed the same experimental protocols again. The time points for exercise capacity and the EMG activity test were set before probiotics were taken (baseline), 48 h before HM (Pre), and immediately at 0 h, 3 h, 24 h, 48 h, 72 h, and 96 h after HM (Figure 1). The exercise capacity test included lower limb strength. EMG activity included MDF, iEMG, and NME (peak torque/iEMG).

This study was conducted according to the guidelines of the Declaration of Helsinki and approved by the Institutional Review Board of the University of Taipei (Protocol number IRB-2020-054 and date of approval: 18 September 2020). All participants gave their informed written consent.

### 2.2 Participants

Eight recreational runners (four males and four females; age: 26.6 ± 1.9 years; height: 166.8 ± 10.4 cm; and weight: 66.6 ± 15.3 kg) were recruited for this study from the University of Taipei according to the following inclusion criteria: a) age 18–25 years; b) no engagement in professional physical training. Exclusion criteria were as follows: a) the presence of known cardiovascular, pulmonary, metabolic, bone, or joint diseases; b) muscle and joint injuries during the last 6 months; and c) use of fermented products (e.g., Yakult or yogurt), minerals, vitamins, antibiotics, and traditional Chinese medicines in the last 6 months. Participants were instructed to continue their regular dietary habits throughout the study period without the use of any performance-enhancing substances or supplements and maintain their daily living without additional exercise. They were informed about the objectives, procedures, and potential risks of this study.

### 2.3 Procedures

#### 2.3.1 *Lactobacillus plantarum* PS128

PS128 was a capsule dosage form manufactured by Bened Biomedical Co., Ltd. [Taipei City, Taiwan (R.O.C)], containing a milky white powder. PS128 is deposited at the DSMZ German Collection of Microorganisms and Cell Cultures, accession number DSM 28632. Each capsule contains 400 mg of lyophilized bacterial powder, which contains 300 mg of lyophilized PS128 powder [1.5 × 10^10^ colony forming units (CFU)] and 100 mg of microcrystalline cellulose as excipients. The placebo group (PLG) took the same type of capsules filled with 400 mg of microcrystalline cellulose twice daily. The PS128 group took one capsule twice daily, equivalent to a dose of 3 × 10^10^ CFU/day. Products were refrigerated at 4–8°C.

#### 2.3.2 Half-marathon

HM was conducted on a 400-m track and field stadium made of polyurethane, situated on the Tianmu campus of the University of Taipei. Participants were required to complete a 5-min jogging session and a 15-min dynamic warm-up before 5 o’ clock in the morning. The distance of HM is 21 km, so the participants should have run 52 laps and 200 m. The first aid station was set near the starting line to offer water or emergency medical care. All the subjects were instructed to fast during HM, with water consumption being the only exception.

#### 2.3.3 Lower limb muscle strength test

The lower limb strength was tested using an isokinetic dynamometer (Biodex System Pro 4; Biodex Medical Systems, Shirley, NY, USA), and the maximal voluntary isometric contraction (MVIC) of knee extensors was measured. Familiarization was executed 2 weeks before the MVIC test. The sitting position was hip flexion at 85° and knee flexion at 30° with the dominant leg, and the chest, hip, and non-dominant thigh were fixed with straps. The participants executed the MVICs of the knee extensor for 3 s, repeating the procedure thrice with a 1-min interval. The best of three attempts was considered.

#### 2.3.4 Electromyographic activity

EMG signals were recorded using myoMUSCLE EMG Analysis Software (myoRESEARCH 3.14, Noraxon, Scottsdale, AZ, United States) to measure the MDF and iEMG of knee extensor muscles, including vastus medialis oblique (VMO), vastus lateralis oblique (VLO), and rectus femoris (RF). Skin preparation involved shaving and cleaning it with alcohol. Wireless EMG sensors, with a 10 mm diameter and 20 mm electrode spacing, were placed on the muscle belly in a bipolar configuration (H. J. Hermens et al., 2000; Hermie J Hermens et al., 1999). The data were sampled at 1,000 Hz. MDF analysis involved fast Fourier transformation (FFT). iEMG was derived through full-wave rectification, smoothed using RMS at 100 Hz, and integrated. NME was calculated as [force/iEMG].

### 2.4 Statistical analysis

All dependent variables were described by the mean ± SD. The data were analyzed using GraphPad Prism software (Version 8; GraphPad Software, San Diego, CA, United States). Statistical significance was set at *p* ≤ 0.05. The normality assumption of the variable was verified using the Shapiro–Wilk test. Homogeneity of variance and sphericity were assessed using Levene’s test and Mauchly’s test, respectively. Then, a two-way repeated-measures analysis of variance (ANOVA) was applied to test the changes in variables over time between the two groups, considering the within-subject factor time (baseline, Pre, 0 h, 3 h, 24 h, 48 h, 72 h, and 96 h). *Post hoc* tests included Tukey’s and pairwise comparisons with Sidak’s adjustments after one-way repeated-measures ANOVA. Paired *t*-tests were used to analyze the performance of HM between the PLG and PSG. All data were normalized by calculating the percentage of the premeasurement (post-test/pre-test) × 100% for comparison. The results were presented as the percentage (%) ± standard deviation (%).

## 3 Results

### 3.1 Test of homogeneity

The results of the test of homogeneity are shown in Table 1. Mean ± SD was used to describe all dependent variables. All statistical tests were conducted using SPSS 20.0 with statistical significance set at *p* ≤ 0.05. The results of the *t*-test show that there was no significant difference between the PLG and PSG at baseline.

**TABLE 1:** Test of homogeneity.

Electromyography activity	Baseline PLG (*n* = 8)	Baseline PSG (*n* = 8)	*p*-value
MDF of VMO	60.81 ± 5.78	55.45 ± 7.30	0.06
MDF of VLO	59.19 ± 5.05	61.19 ± 6.47	0.29
MDF of RF	63.45 ± 5.77	66.38 ± 10.65	0.30
iEMG of VMO	750.88 ± 312.29	750.25 ± 415.63	0.50
iEMG of VLO	796.00 ± 283.30	665.25 ± 279.94	0.29
iEMG of RF	828.13 ± 287.86	897.75 ± 381.98	0.24
NME of VMO	0.35 ± 0.21	0.33 ± 0.22	0.41
NME of VLO	0.30 ± 0.10	0.32 ± 0.12	0.41
NME of RF	0.30 ± 0.15	0.23 ± 0.03	0.09

MDF, median power frequency; VMO, vastus medialis oblique; VLO, vastus lateralis oblique; RF, rectus femoris; iEMG, integrated electromyography; HM, half-marathon; PLG, placebo group; PSG, *Lactobacillus plantarum* PS128 group; NME, neuromuscular efficiency. * indicates a significant difference between the PLG and PSG. *p* < 0.05 indicates a statistically significant difference.

### 3.2 Half-marathon

**FIGURE 2:**
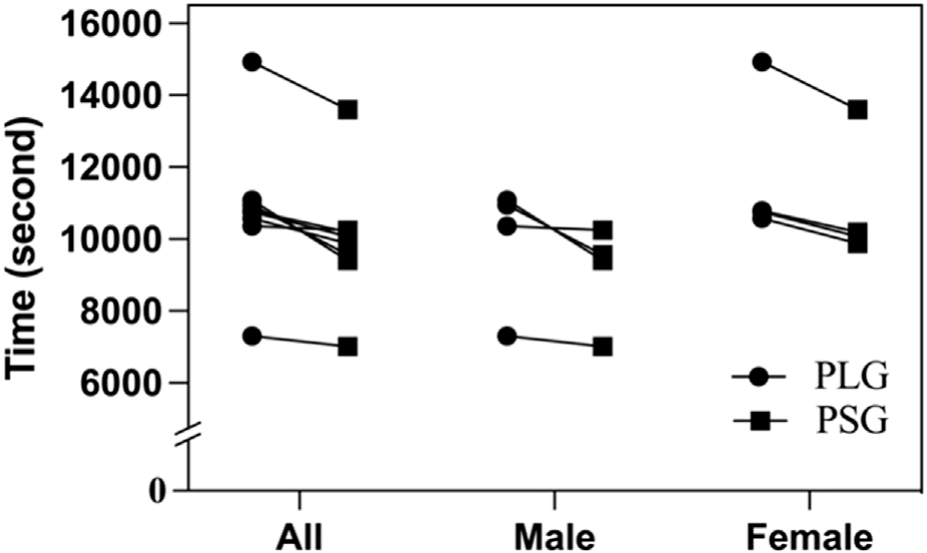
Accomplished time of half-marathon. PLG, placebo group; PSG, *Lactobacillus plantarum* PS128 group.

The environmental conditions of the first trial to HM were a temperature of 17.1°C–18.6°C (05:00 a.m.–09:00 a.m.), wind speed of 1.0–1.1 m/s, and relative humidity of 81%–84%. The second trial was executed after a 3-month washout period, and the nutritional supplements were exchanged in two groups. The environmental conditions were a temperature of 17.5°C–18.9°C (05:00 a.m.–09:00 a.m.), wind speed of 1.2–1.3 m/s, and relative humidity of 76%–79%. The accomplished time of HM by the PLG varied from 02:01:40 to 04:08:52 (HH:MM:SS), with a mean duration of 03:00:43 ± 00:34:18 (02:45:24 ± 00:29:37 in males and 03:16:03 ± 00:35:15 in females). The accomplished time of HM by the PSG varied from 01:56:55 to 03:46:42, with a mean duration of 02:46:36 ± 00:29:55 (02:30:59 ± 00:23:31 in males and 03:02:14 ± 00:29:43 in females). The *t*-tests showed no significant difference between the PLG and PSG in HM (*p* = 0.198) (males in the PLG and PSG: *p* = 0.238; females in the PLG and PSG: *p* = 0.286) (Figure 2).

### 3.3 Electromyography activity

**FIGURE 3:**
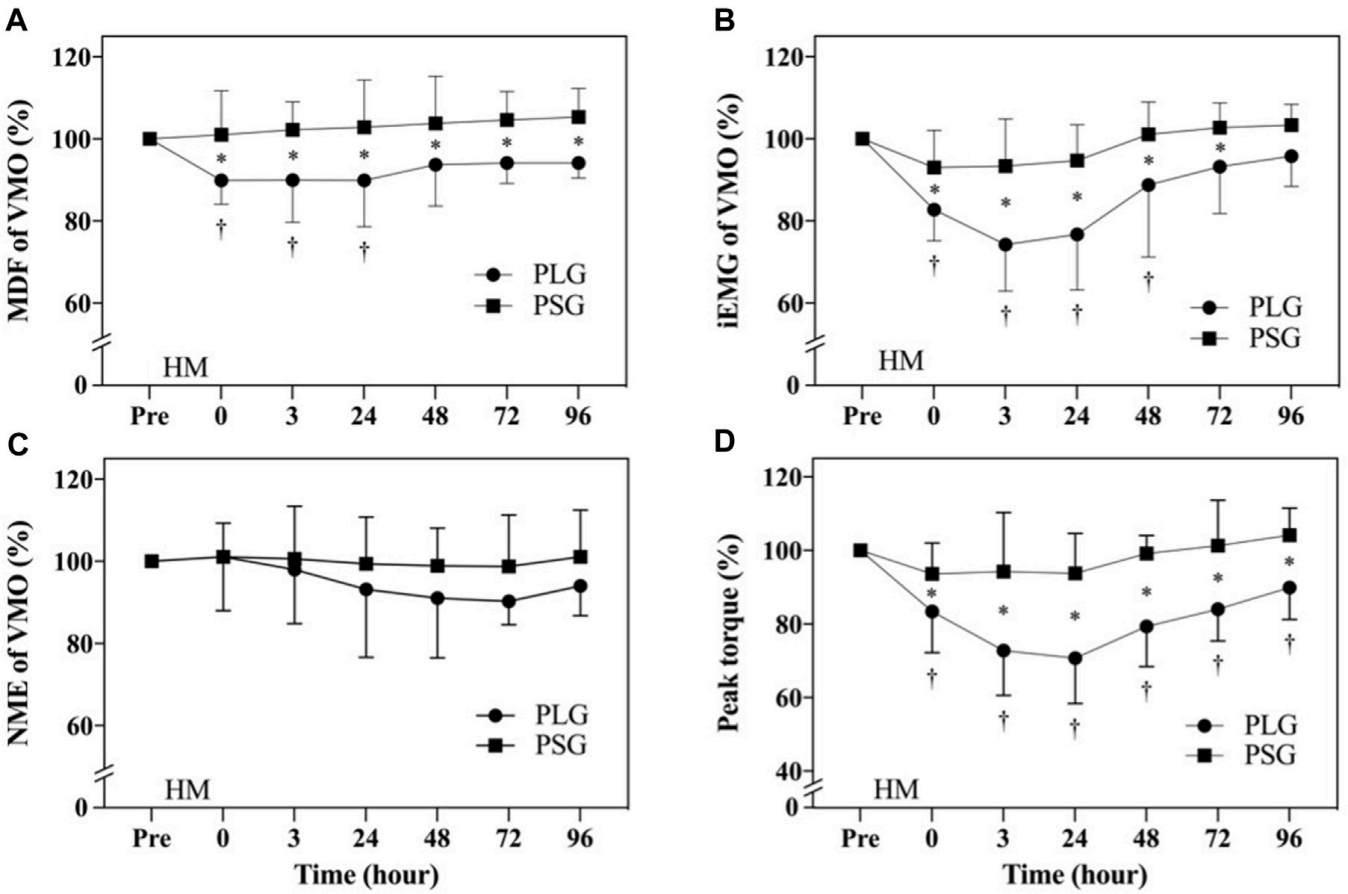
Comparison of electromyography of vastus medialis oblique and lower limb muscle strength. The data are represented as the mean ± standard deviation. ● represents the placebo group; ■ represents the PS128 group. **(A)** MDF of VMO; **(B)** iEMG of VMO; **(C)** NME of VMO; and **(D)** peak torque of the MVIC of knee extensors. HM, half-marathon; † indicates a significant change from Pre to post time points in the PLG; and * indicates a significant difference between the PLG and PSG at the same time point. *p* < 0.05 indicates a statistically significant difference within and between groups.

Descriptive statistics (mean ± SD) for the investigated variables are presented in Table 2. According to the aforementioned results, significant differences were found only in VMO. Individual variation should be avoided so that all data are normalized by calculating the percentage of the premeasurement (post-test/pre-test) × 100% for comparison. The results were shown as the percentage (%) ± standard deviation (%). Figures 3A–C show the results of the MDF, iEMG, and NME of VMO. The results from a two-way repeated-measures ANOVA presented a significant time-by-group interaction for the MDF of VMO (*p* = 0.042) and the iEMG of VMO (*p* = 0.0012). After that, Tukey’s multiple comparisons tests showed that the MDF of VMO decreased from the Pre to 0 h (*p* = 0.0144), 3 h (*p* = 0.0163), and 24 h (*p* = 0.0144) time points in the PLG (Figure 3A); the iEMG of VMO decreased from the Pre to 0 h (*p* < 0.0001), 3 h (*p* < 0.0001), 24 h (*p* < 0.0001), and 48 h (*p* = 0.0101) time points in the PLG (Figure 3B). Sidak’s multiple comparisons tests showed that the MDF of VMO for the PLG was significantly lower than that for the PSG at 0 h post (*p* = 0.0022), 3 h post (*p* = 0.0006), 24 h post (*p* = 0.0003), 48 h post (*p* = 0.0071), 72 h post (*p* = 0.0042), and 96 h post (*p* = 0.0019) time points (Figure 3A); the iEMG of VMO for the PLG was significantly lower than that for the PSG at 0 h post (*p* = 0.0111), 3 h post (*p* < 0.0001), 24 h post (*p* < 0.0001), 48 h post (*p* = 0.0014), and 72 h post (*p* = 0.0221) time points (Figure 3B). The NME of VMO in the PLG and PSG showed no significant difference after HM (Figure 3C).

**TABLE 2:** Mean ± SD for the MDF of VMO, VLO, and RF; iEMG of VMO, VLO, and RF; and NME of VMO, VLO, and RF, at baseline, 48 h before (Pre), and 0 h, 3 h, 24 h, 48 h, 72 h, and 96 h after HM for the PLG and PSG.

	Baseline	Pre	0 h	3 h	24 h	48 h	72 h	96 h
MDF of VMO			†*	†	†			
PLG	60.81 ± 5.78	61.03 ± 5.98	54.63 ± 2.66	54.63 ± 2.66	54.34 ± 4.53	56.78 ± 4.77	57.29 ± 4.64	54.63 ± 2.66
PSG	55.45 ± 7.30	55.36 ± 7.63	55.50 ± 6.66	56.55 ± 7.80	56.40 ± 5.25	56.85 ± 4.63	57.83 ± 8.76	58.03 ± 6.10
MDF of VLO								
PLG	59.19 ± 5.05	59.25 ± 5.24	60.19 ± 5.50	60.03 ± 6.07	61.43 ± 7.53	61.96 ± 6.60	62.45 ± 5.14	62.10 ± 4.05
PSG	61.19 ± 6.47	61.28 ± 6.31	58.98 ± 6.99	60.09 ± 7.99	57.35 ± 6.80	57.45 ± 3.82	57.58 ± 10.13	62.35 ± 5.71
MDF of RF								
PLG	63.45 ± 5.77	63.53 ± 5.74	65.16 ± 8.96	63.34 ± 5.72	63.53 ± 8.65	65.63 ± 7.77	66.28 ± 10.76	64.74 ± 8.07
PSG	66.38 ± 10.65	66.59 ± 10.73	65.36 ± 10.23	64.21 ± 13.15	63.54 ± 8.28	65.20 ± 11.21	64.36 ± 10.39	65.33 ± 13.28
iEMG of VMO			†	†*	†*	†*	*	
PLG	750.88 ± 312.29	751.13 ± 321.97	616.13 ± 252.12	550.13 ± 219.55	563.25 ± 211.54	651.38 ± 246.53	692.00 ± 281.89	706.75 ± 270.41
PSG	750.25 ± 415.63	750.38 ± 419.59	688.13 ± 357.73	700.13 ± 377.13	703.75 ± 379.21	755.75 ± 403.15	770.25 ± 415.85	772.00 ± 411.99
iEMG of VLO								
PLG	796.00 ± 283.30	794.00 ± 281.47	768.00 ± 282.11	693.13 ± 418.97	783.88 ± 267.67	910.38 ± 492.08	980.63 ± 544.82	1,084.50 ± 558.79
PSG	665.25 ± 279.94	665.75 ± 278.48	735.38 ± 438.18	691.75 ± 432.36	843.50 ± 427.00	872.50 ± 477.92	927.88 ± 445.32	910.13 ± 528.12
iEMG of RF								
PLG	828.13 ± 287.86	827.63 ± 287.96	701.25 ± 235.22	612.38 ± 301.80	679.25 ± 200.47	752.75 ± 293.59	794.88 ± 332.49	842.50 ± 288.85
PSG	897.75 ± 381.98	884.13 ± 403.84	903.75 ± 522.20	720.38 ± 380.56	956.50 ± 560.28	953.50 ± 519.44	1,029.88 ± 646.93	970.13 ± 460.85
NME of VMO								
PLG	0.35 ± 0.21	0.35 ± 0.22	0.35 ± 0.18	0.33 ± 0.15	0.31 ± 0.14	0.31 ± 0.16	0.31 ± 0.18	0.33 ± 0.19
PSG	0.33 ± 0.22	0.32 ± 0.23	0.32 ± 0.21	0.31 ± 0.21	0.32 ± 0.25	0.31 ± 0.20	0.31 ± 0.22	0.32 ± 0.22
NME of VLO								
PLG	0.30 ± 0.10	0.30 ± 0.11	0.26 ± 0.08	0.29 ± 0.17	0.20 ± 0.05	0.22 ± 0.08	0.23 ± 0.09	0.21 ± 0.08
PSG	0.32 ± 0.12	0.32 ± 0.13	0.28 ± 0.12	0.27 ± 0.07	0.25 ± 0.09	0.24 ± 0.07	0.22 ± 0.07	0.25 ± 0.08
NME of RF								
PLG	0.30 ± 0.15	0.30 ± 0.16	0.28 ± 0.09	0.46 ± 0.39	0.36 ± 0.27	0.36 ± 0.15	0.26 ± 0.08	0.25 ± 0.08
PSG	0.23 ± 0.03	0.23 ± 0.04	0.21 ± 0.03	0.26 ± 0.06	0.22 ± 0.05	0.22 ± 0.03	0.21 ± 0.05	0.22 ± 0.04

MDF, median power frequency; VMO, vastus medialis oblique; VLO, vastus lateralis oblique; RF, rectus femoris; iEMG, integrated electromyography; HM, half-marathon; PLG, placebo group; PSG, *Lactobacillus* plantarum PS128 group; NME, neuromuscular efficiency. † indicates a significant change from Pre to post time points in the PLG; * indicates a significant difference between the PLG and PSG at the same time point. *p* < 0.05 indicates a statistically significant difference within and between groups.

### 3.4 Analysis of lower limb muscle strength

The MVICs of knee extensors analyzed using an isokinetic dynamometer are shown in Figure 3D. The results from a two-way repeated-measures ANOVA showed a significant time-by-group interaction (*p* < 0.0001). After that, Tukey’s multiple comparisons tests showed a decrease from the Pre to 0 h (*p* = 0.0001), 3 h (*p* < 0.0001), 24 h (*p* < 0.0001), 48 h (*p* < 0.0001), 72 h (*p* = 0.0002), and 96 h (*p* = 0.0408) time points in the PLG. Sidak’s multiple comparison tests showed that the PLG was significantly lower than the PSG at 0 h (*p* = 0.0173), 3 h (*p* < 0.0001), 24 h (*p* < 0.0001), 48 h (*p* < 0.0001), 72 h (*p* < 0.0001), and 96 h (*p* = 0.0004) time points.

## 4 Discussion

This study aimed to continue the research results of the effect of daily oral PS128 on exercise capacity recovery after HM mentioned by Fu Szu-Kai et al. (2021) and confirmed with different evidence. Therefore, this study investigated the effect of PS128 on muscle fatigue and EMG activity after HM. The eight subjects in this study differed from those in the study by Fu Szu-Kai (2021). This study aimed to contribute to scientific knowledge regarding the effectiveness of PS128 on muscle fatigue and EMG activity to find out the underlying mechanisms. We hypothesized that taking PS128 could prevent the decrease in NME, iEMG, and MVIC on knee extensors after HM. In addition, we expected that the decrease in MDF could be prevented. The results of this study support the hypothesis that there was no significant decrease in MDF, iEMG, and MVIC in the PSG compared to the PLG. Recreational runners might consider adopting a probiotic supplementation protocol as a potential strategy to mitigate muscle fatigue following HM.

After HM, it was found that the MDF, iEMG, and MVIC of VMO in the PLG exhibited a significant downward trend. MDF showed a frequency decline, indicating reduced fast-twitch muscle recruitment (Wang et al., 2015). One potential contributing factor might be the heightened release of potassium ions (K+) from muscle fibers. Elevated potassium levels within the T-tubules could impede the tubular action potential, resulting in diminished force production due to a disruption in excitation–contraction coupling (Ament and Verkerke, 2009). During running, the quadriceps play a crucial role in decelerating knee flexion in the standing phase. VLO and VMO worked together in the late swing phase to extend the knee and stabilize the patella and knee during running. They also contribute to decelerating knee flexion in the early and middle swing phases. RF collaborates with the vastus intermedius (Montgomery et al., 1994). RF contracts during the early and middle swing phases to facilitate hip flexion and control knee flexion (Montgomery et al., 1994). It is worth noting that the optimal joint angle for VMO output is between 0 and 30° (Bagheri et al., 2005), while VLO output is optimal at 90° (Marchetti et al., 2016). The running movement pattern, with a knee joint angle of approximately 20° (Petit et al., 2013), might lead to greater fatigue in VMO but not VLO. The decrease in MDF in the PLG reflected reduced recruitment of fast-twitch muscles, and there was a significant decrease in the total amount of iEMG, which could affect exercise performance.

In addition to the physiological changes associated with peripheral fatigue, there are metabolic, neuromuscular, and mechanical implications for muscle cells stemming from fatigue. These can be attributed to three primary factors: energy metabolism failure, inefficiency in excitation–contraction coupling, and metabolic acidosis. Energy metabolism failure occurs when the muscle consumes adenosine triphosphate (ATP) at a faster rate than it can regenerate. As muscle phosphocreatine stores deplete, there is an increase in muscle creatine and circulating phosphates. Consequently, levels of adenosine diphosphate (ADP) rise, as do concentrations of adenosine monophosphate (AMP). AMP is metabolized by AMP deaminase, becoming phosphate-free and capable of binding to ADP molecules to form ATP, thereby supplying the muscle tissue (Allen et al., 2008). The excitation–contraction coupling mechanism is affected in various ways. First, damage occurs, particularly during eccentric contractions, to the protein junctophilin, which plays a role in the connection between the T-tubule and sarcoplasmic reticulum (McKenna and Hargreaves, 2008). The metabolic acidosis is related to the accumulation of Pi and H+, which has a negative effect on the affinity of the thin filaments with calcium, directly reducing its release from the sarcoplasmic reticulum and, therefore, its *mycoplasma* concentration, with the consequent reduction in force production (Debold et al., 2016).

Encarnación-Martínez et al. (2022) organized a study involving 18 male recreational runners who ran on a treadmill for 30 min at a 0% slope, maintaining a pace corresponding to 85% of their maximal aerobic speed, which led to CNS fatigue. Boccia et al. (2018) conducted an HM intervention with 21 amateur runners, comprising 11 males and 10 females. The results indicated moderate CNS fatigue and minor peripheral fatigue. The NME of the PLG and PSG had no significant differences after the HM intervention because the peak torque of the PLG had a similar downward trend after the decrease in iEMG. Such results could infer the absence of peripheral fatigue in the two groups. The intervention of long-distance running would cause CNS fatigue, but peripheral fatigue needed to be induced in the form of higher-intensity resistance exercise. For example, Soleimanifar et al. (2012) executed 120°/s of continuous concentric/concentric knee flexion and extension during a peripheral fatigue intervention in 24 healthy young women, maximizing strength throughout the range of motion without rest. The fatigue protocol ends when the concentric peak torque falls below 50% for three consecutive times in both directions. Given the well-established association between long-distance running and CNS fatigue, the absence of CNS fatigue in the PSG might be attributed to the PS128 intervention, known for its antioxidative benefits.

Significant elevations in ROS levels following intense exercise could result in contractile dysfunction and muscle atrophy, contributing to muscle weakness and fatigue (Reid, 2008). Another issue arising from the increased ROS levels was the damage inflicted upon mitochondria. It had been demonstrated that increased ROS levels could cause severe damage to the membranes of mitochondria, consequently reducing mitochondrial biogenesis and, in turn, diminishing ATP production (García-Mesa et al., 2015). Enhancements in anti-inflammatory, antioxidant, and metabolic functions mitigated the detrimental effects of high-intensity sports on exercise capacity. High-intensity exercise could lead to muscle damage, disrupt calcium balance, trigger neutrophil infiltration, and promote the generation of free radicals and cytohormone metabolites. Consequently, this results in elevated levels of inflammatory markers, creatine phosphokinase (CPK) and lactate dehydrogenase (LDH), and oxidative stress-related markers, superoxide dismutase (SOD) and catalase (CAT) (Withee et al., 2017). PS128 probiotics could reduce muscle damage indices (such as myoglobin and CPK) after HM, showing that nutritional supplements positively affect muscle damage, elevate the antioxidation indicator SOD, and enhance exercise capacity (Fu et al., 2021). The positive impact of PS128 on oxidative stress, inflammation, and performance in high-intensity exercise for triathletes was demonstrated by Huang et al. (2019). Teichoic acids (TAs) produced by *Lactobacillus plantarum* play a crucial role in systematically regulating the immune response in mice (W.-H. Liu et al., 2015). PS128 reduced the production of pro-inflammatory cytokines and positively regulated inflammation, oxidation, and metabolism during high-intensity exercise in a mouse macrophage model. This was further supported by its impact on myoglobin, LDH, CPK, and SOD levels. Both clinical and non-clinical studies have confirmed the anti-inflammatory properties of *Lactobacillus plantarum* and its ability to modulate the host’s immune response.

CNS fatigue could be explained by a reduced firing rate of the motor neuron pool at the spinal, which attenuates signals for muscle activation (Boyas et al., 2013). Therefore, both CNS and peripheral fatigue affected muscle force production. It was a potential factor obtained by analyzing the power spectrum of the EMG signal in the experiment protocol, which could objectively identify muscle fatigue.

## 5 Conclusion

The PS128 supplementation might prevent the decrease in MDF, iEMG, and peak torque after HM. Consequently, recreational runners might contemplate the adoption of a probiotic supplementation protocol as a prospective strategy to mitigate muscle fatigue after HM. It was advised that recreational runners consider a 4-week probiotic supplementation regimen to mitigate post-HM muscle fatigue.

## Data Availability

The original contributions presented in the study are included in the article/Supplementary material; further inquiries can be directed to the corresponding author.
